# A Single Amino Acid Substitution in the Novel H7N9 Influenza A Virus NS1 Protein Increases CPSF30 Binding and Virulence

**DOI:** 10.1128/JVI.01567-14

**Published:** 2014-10

**Authors:** Juan Ayllon, Patricia Domingues, Ricardo Rajsbaum, Lisa Miorin, Mirco Schmolke, Benjamin G. Hale, Adolfo García-Sastre

**Affiliations:** a Department of Microbiology, Icahn School of Medicine at Mount Sinai, New York, New York, USA; b Department of Medicine, Division of Infectious Diseases, Icahn School of Medicine at Mount Sinai, New York, New York, USA; c Global Health and Emerging Pathogens Institute, Icahn School of Medicine at Mount Sinai, New York, New York, USA; d MRC—University of Glasgow Centre for Virus Research, Glasgow, United Kingdom

## Abstract

Although an effective interferon antagonist in human and avian cells, the novel H7N9 influenza virus NS1 protein is defective at inhibiting CPSF30. An I106M substitution in H7N9 NS1 can restore CPSF30 binding together with the ability to block host gene expression. Furthermore, a recombinant virus expressing H7N9 NS1-I106M replicates to higher titers *in vivo*, and is subtly more virulent, than the parental virus. Natural polymorphisms in H7N9 NS1 that enhance CPSF30 binding may be cause for concern.

## TEXT

Since early 2013, zoonotic transmission of a novel avian-origin H7N9 influenza A virus in eastern China has led to at least 441 human infections and 122 deaths (1). Sequence analyses and functional studies have identified several mammalian adaptive polymorphisms in PB2 (2, 3), PA (4), hemagglutinin (HA), and neuraminidase (NA) (5, 6) that may be responsible for promoting replication and pathogenicity of this virus in humans. Nevertheless, it is clear that avian-origin H7N9 has yet to adapt to humans: HA retains its preference for avian receptors (7–10), and H7N9 is unable to transmit efficiently between humans. Identifying sequence changes in H7N9 that may evolve naturally and contribute to future human-to-human transmission, or that may alter H7N9 virulence, is critical.

### Novel H7N9 virus NS1 is an IFN antagonist in human and chicken cells.

**FIG 1:**
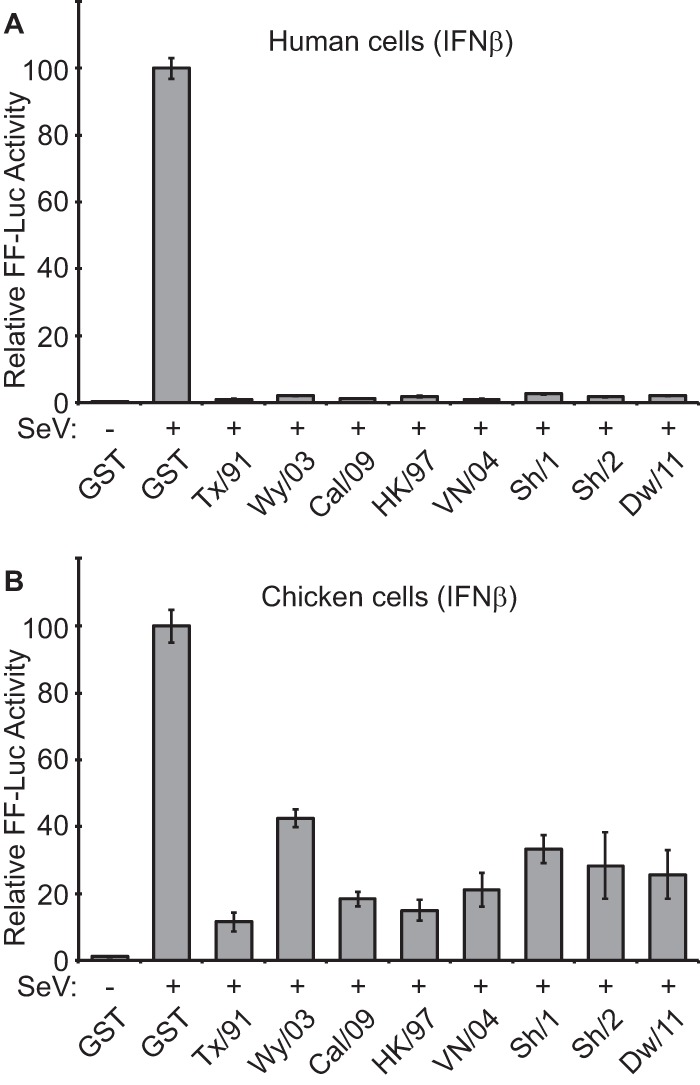
The H7N9 NS1 protein is an IFN antagonist in human and chicken cells. Human 293T (A) or chicken DF-1 (B) cells were cotransfected for 16 h with a pCAGGS expression plasmid encoding the indicated NS1 protein (or GST) together with a FF luciferase (FF-Luc) IFN-β promoter reporter plasmid (p125Luc). After infection with a DI-rich SeV preparation for a further 12 h, FF-Luc activity was determined. Results represent the means and standard deviations of triplicate values (normalized to GST + SeV) obtained in a single experiment and are representative of results of two independent experiments. The NS1 sequences (containing silent splice acceptor mutations to prevent NEP/NS2 expression [12]) were derived from A/Texas/36/1991 (Tx/91; human seasonal-like H1N1 virus), A/Wyoming/03/2003 (Wy/03; human seasonal-like H3N2), A/California/04/2009 (Cal/09; human seasonal-like H1N1, previously 2009 pdmH1N1), A/Hong Kong/156/1997 (HK/97; representative of the 1997 H5N1 outbreak), A/Vietnam/1203/2004 (VN/04; representative of the 2004 H5N1 outbreak), A/Shanghai/1/2013 (Sh/1; human H7N9), A/Shanghai/2/2013 (Sh/2; human H7N9), and A/Chicken/Dawang/1/2011 (Dw/11; avian H9N2 with NS1 closely related to H7N9 NS1).

NS1 is a multifunctional virulence factor that plays a major role in antagonizing host interferon (IFN) responses during infection (reviewed in reference 11). Notably, this property can vary in efficiency between virus isolates and between the species of origin of the host cell (12–16). We tested the ability of H7N9 NS1 to limit production of beta IFN (IFN-β) in human and avian cells and compared the results to those from a panel of NS1 proteins from seasonal human influenza viruses or other avian H5N1 viruses that have sporadically infected humans since 1997 (Fig. 1). Human 293T, or chicken DF-1, cells were transfected with an IFN-β promoter-driven firefly luciferase (FF-Luc) reporter construct together with expression plasmids for the NS1 proteins of interest (or glutathione *S*-transferase [GST] as a control). For analysis of novel H7N9, we tested NS1 proteins derived from two different human H7N9 strains (A/Shanghai/1/2013 [Sh/1] and A/Shanghai/2/2013 [Sh/2]), along with the NS1 protein from a closely related avian H9N2 virus, A/chicken/Dawang/1/2011 (Dw/11). Subsequent infection with a defective interfering genome (DI)-rich Sendai virus (SeV) preparation induced robust amounts of IFN-β promoter-driven FF-Luc activity in GST-expressing human 293T cells but not in cells expressing any of the NS1 proteins (Fig. 1A). Similar results were observed for chicken DF-1 cells, although the inhibitory impact of all NS1s in this system was less evident (Fig. 1B), possibly due to the absence of chicken RIG-I (17), a key target for NS1 in humans (18). Notably, A/Texas/36/1991 (Tx/91) NS1, a well characterized and potent inhibitor of general host gene expression (14), appeared to act as the strongest IFN antagonist. These data suggest that the human H7N9 and avian H9N2 precursor NS1s can function as IFN antagonists in human and chicken cells, a finding in agreement with recent results from others (19).

### A single I106M substitution in novel H7N9 NS1 enhances CPSF30 binding and inhibition of host gene expression.

**FIG 2:**
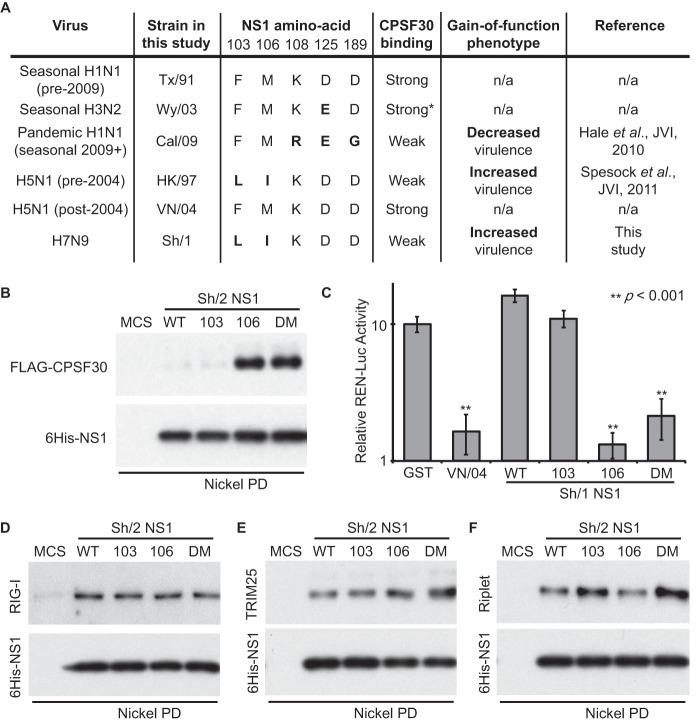
A single I106M substitution in the H7N9 NS1 protein specifically restores efficient CPSF30 binding and inhibition of host cell gene expression. (A) Summary data for a selection of NS1s used in this study. The asterisk denotes predicted binding affinity based on related H3N2 viruses. (B) Binding of NS1 to CPSF30. 293T cell lysate overexpressing FLAG-CPSF30 was mixed with the indicated bacterially expressed 6His-NS1 protein (WT, L103F, I106M, or L103F/I106M [DM] or the 6His multiple cloning site [6His-MCS] negative control) and pulled down (PD) using Ni-nitrilotriacetic acid (NTA) beads. Precipitates eluted after extensive washing were analyzed by SDS-PAGE and Western blotting using anti-NS1 and anti-FLAG antibodies. (C) NS1-mediated inhibition of general host gene expression. Human 293T cells were cotransfected with a pCAGGS expression plasmid encoding the indicated NS1 protein (or GST) together with a constitutively active Renilla-luciferase plasmid. Luciferase activity was determined 24 h posttransfection. Results are given as the means and standard deviations of triplicate values normalized to GST. Statistical significance (**) was determined using the Student *t* test. (D to F) Binding of NS1 to cellular factors involved in the IFN induction cascade. Experiments were performed as for panel B, using 293T lysates overexpressing FLAG-RIG-I (D), V5-TRIM25 (E), or HA-Riplet (F). Western blotting was performed using appropriate anti-tag antibodies.

Binding of NS1 to cellular CPSF30 contributes to the global posttranscriptional inhibition of cellular pre-mRNA processing during infection and is an additional mechanism by which most human influenza viruses attenuate host antiviral responses (20–22). This property of NS1 is not conserved in all strains (14, 23), and variation in CPSF30 binding has been reported to arise when viruses are adapted to replicate in certain new host species (24, 25). There are two notable examples of naturally occurring influenza viruses that have infected humans but which encode NS1 proteins unable to inhibit CPSF30: highly pathogenic avian-origin 1997 H5N1 virus (23) and the swine-origin 2009 pandemic H1N1 (pdmH1N1) virus (12). The defect in CPSF30 binding maps to slightly different amino acid positions in the 1997 H5N1 and 2009 pdmH1N1 NS1 proteins (summarized in Fig. 2A), and opposite phenotypes have been identified for “gain-of-function” substitutions that restore CPSF30 inhibition in these two viruses: for 1997 H5N1, gain of CPSF30 binding promotes systemic spread of the virus and increases virulence (26), while for 2009 pdmH1N1, gain of CPSF30 binding slightly decreases replication and virulence (12). Such phenotypic differences may be due to the disparate amino acid substitutions required to restore binding or to the distinct genetic constellations of the two avian- or swine-origin viruses. Certainly, the impact on replication and virulence of modulating CPSF30 binding affinity is complex and is seemingly unpredictable between strains (12, 24–28).

Intriguingly, we observed that the novel H7N9 NS1 protein has the same amino acid polymorphisms that weaken CPSF30 binding as the 1997 H5N1 NS1 protein (Fig. 2A). We tested the coprecipitation of FLAG-tagged CPSF30 with bacterially expressed 6His-tagged wild-type (WT) Sh/2 NS1, as well as with L103F, I106M, or L103F/I106M double-mutant (DM) variants that we predicted might have a different CPSF30-binding profile. Only a small amount of FLAG-CPSF30 could be precipitated by NS1-WT or NS1-L103F (Fig. 2B). However, FLAG-CPSF30 bound efficiently (and equally) to NS1-I106M and NS1-DM, suggesting that the H7N9 NS1-CPSF30 interaction is indeed weak but can be strengthened by the single I106M substitution.

To test the ability of H7N9 NS1 to block general host gene expression, we cotransfected 293T cells with a constitutively active Renilla luciferase reporter construct (pRL-SV40) together with each of the indicated Sh/1 NS1 constructs and measured total Renilla luciferase activity 24 h later. As shown in Fig. 2C, the WT H5N1 A/Vietnam/1203/2004 (VN/04) NS1 protein (that binds CPSF30 [23]) efficiently inhibited Renilla luciferase activity, while the H7N9 NS1-WT protein (like GST) was deficient in this function. In agreement with our pulldown studies, NS1-L103F was also unable to inhibit Renilla luciferase activity, while both NS1-I106M and NS1-DM proteins efficiently limited activity (Fig. 2C). These results indicate that the single I106M substitution in H7N9 NS1 restores both CPSF30 binding and the inhibition of cellular gene expression. The I106M substitution specifically enhanced CPSF30 binding, as no differences were observed between NS1-WT and any of the NS1 mutants with regard to interaction with RIG-I (18) or TRIM25 (29) (Fig. 2D and E). The I106M substitution also did not have an impact on the coprecipitation of Riplet with NS1 (16), although there was a possible enhancement of this interaction when NS1 constructs contained the L103F substitution (Fig. 2F). These data highlight a key difference with the 1997 H5N1 NS1 protein, where a similar substitution at position 106 was reported to differentially affect CPSF30 and RIG-I binding (27).

### Characterization of an H7N9-based virus expressing NS1-I106M *in vitro*.

**FIG 3:**
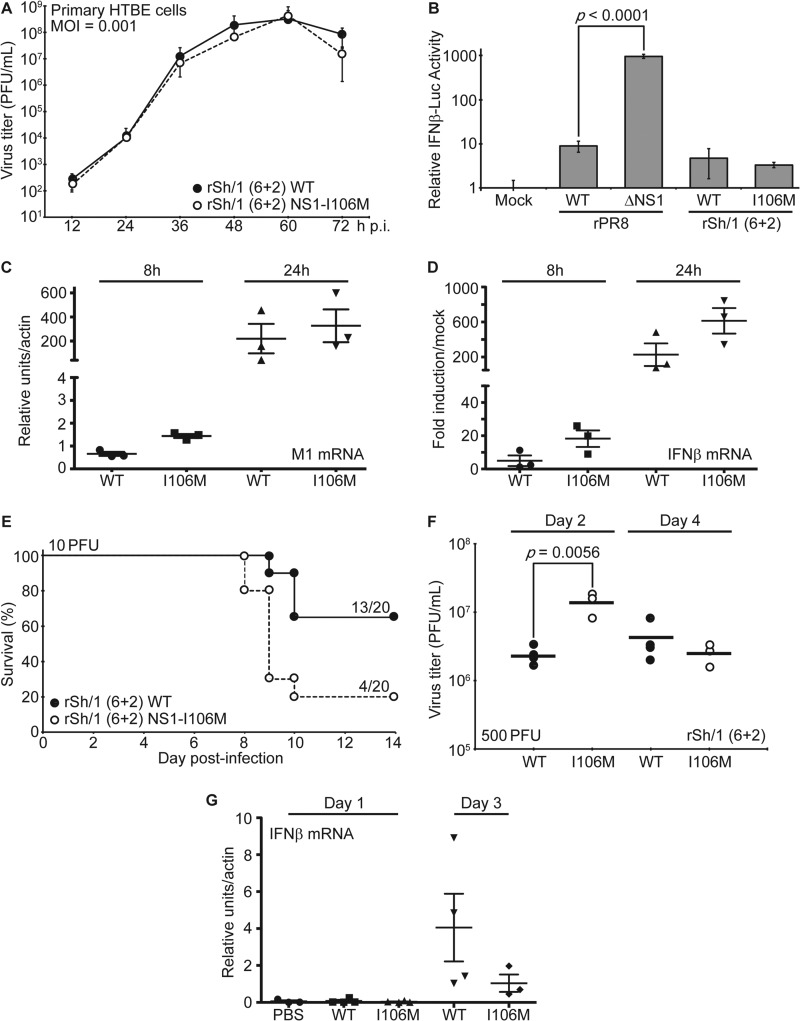
Characterization of H7N9-based NS1-WT and NS1-I106M viruses *in vitro* and *in vivo*. (A) Virus replication *in vitro*. Multicycle growth analysis of rSh/1 (6+2) WT and rSh/1 (6+2) NS1-I106M viruses in primary differentiated human airway epithelial (HTBE) cells. Data points show mean values, and error bars represent standard deviations. (B) Induction of IFN-β by the recombinant viruses. MDCK–IFN-β–FF-Luc cells were infected at a multiplicity of infection (MOI) of 2 PFU/cell for 16 h with the indicated virus (or were mock infected) prior to analysis of luciferase activity. Bars represent mean values (*n* = 3), and error bars represent standard deviations. (C and D) qRT-PCR analyses of viral replication and IFN-β induction *in vitro*. Primary HTBE cells were infected at an MOI of 2 PFU/cell and lysed at the times indicated. Total RNA was extracted, and following reverse transcription using oligo(dT), the levels of viral M1 mRNA (C) and IFN-β mRNA (D) were quantified in triplicate by qPCR. Values were averaged and normalized to actin mRNA. Mean induction levels relative to mRNA levels in mock-infected cells are shown. Error bars represent standard deviations. Means and standard deviations were calculated from biological triplicates. (E) Virulence in mice. Survival data for 6- to 8-week-old C57/BL6 mice infected intranasally with 10 PFU of the indicated virus (20 mice per virus). Body weights were determined daily for 14 days, and mice showing more than 25% weight loss were considered to have reached the experimental endpoint and were humanely euthanized. (F) Virus replication *in vivo*. Six- to eight-week-old C57/BL6 mice were infected intranasally with 500 PFU of the indicated virus. Lung titers were determined on days 2 and 4 postinfection from 3 to 4 mice per group. Bars represent mean values. Statistical significance was determined using the Student *t* test. (G) qRT-PCR analyses of IFN-β induction *in vivo*. Six-to-eight week-old C57/BL6 mice (3 to 4 mice per group) were infected intranasally with 500 PFU of the indicated virus for 1 or 3 days. Murine IFN-β mRNA was quantified from lung homogenates by qRT-PCR as described for panel D. PBS, phosphate-buffered saline.

Using an H7N9 reverse genetics system (30), we generated recombinant WT and NS1-I106M viruses containing the 6 “internal” gene segments of Sh/1 and the HA and NA gene segments from A/Puerto Rico/8/1934 (PR8; H1N1). This “6+2” strategy using the surface antigens of the laboratory PR8 H1N1 strain was adopted for risk mitigation purposes given the gain-of-function nature of the experiment. The two viruses [rSh/1 (6+2) WT and rSh/1 (6+2) NS1-I106M, where “r” indicates recombinant] were plaque purified, and stocks were grown and titrated in MDCK cells. The NS genomic segment of each virus from stock aliquots was sequenced to ensure the absence of additional mutations. Surprisingly, multicycle replication experiments revealed that the recombinant WT and NS1-I106M viruses grew with kinetics identical to each other in primary differentiated human tracheobronchial epithelial (HTBE) cells (Clonetics, Lonza, Walkersville, MD, USA) (Fig. 3A). We also assessed the ability of the two viruses to repress IFN-β production by using an MDCK cell line stably expressing FF-Luc under the control of the IFN-β promoter (31). Notably, both WT and NS1-I106M virus infection led to the same low-level (∼3- to 5-fold) induction of the reporter (Fig. 3B). The similarity of replication kinetics and IFN-β induction levels for the two viruses in primary HTBE cells was independently confirmed by quantitative real-time PCR (qRT-PCR) (Fig. 3C and D). These data suggest that in the context of rSh/1 (6+2), the defect in efficient CPSF30 binding by H7N9 NS1 does not significantly affect virus replication in primary human cells or virus-mediated IFN-β antagonism *in vitro*. This may be due to the impact of other IFN antagonists encoded by the virus (reviewed in reference 32) or the observation that H7N9 NS1 is an efficient IFN antagonist (19) and retains affinity for RIG-I/TRIM25/Riplet. It is also possible that partial compensation of the H7N9 NS1-CPSF30 deficiency occurs by the cognate H7N9 viral polymerase complex, as has been shown for 1997 H5N1 (23, 33).

### An H7N9-based virus expressing NS1-I106M shows enhanced replication and virulence *in vivo*.

To test whether increased affinity of H7N9 NS1 toward CPSF30 would affect viral replication and pathogenicity *in vivo*, we determined 50% mouse lethal dose (MLD_50_) values for the WT and NS1-I106M viruses in 6- to 8-week-old C57BL/6 mice (Jackson Laboratory, ME). All procedures were performed in accordance with the IACUC guidelines of Icahn School of Medicine at Mount Sinai, and animals showing >25% weight loss were considered to have reached the experimental endpoint and were humanely euthanized. MLD_50_ values were subsequently calculated according to the method of Reed and Muench (the data are summarized in Table 1). Even in the highly virulent rSh/1 (6+2) background, the NS1-I106M mutation led to a modest ∼2.5-fold increase in MLD_50_, and mice infected with the NS1-I106M virus exhibited greater overall mortality (Fig. 3E) and morbidity (as determined by the duration and extent of weight loss and the day of death; Table 1) than mice infected with the WT virus. We speculate that the virulence-enhancing impact of H7N9 NS1-I106M may be even more pronounced in the context of non-PR8 glycoproteins, where the respective WT virus would have a much higher MLD_50_.

**TABLE 1:** Characterization of H7N9-based NS1-WT and NS1-I106M viruses *in vivo*^*a*^

Virus [rSh/1 (6 + 2)]	MLD_50_ (PFU)	Dose (PFU)	% survival (no. of survivors/total no. tested)	Median day of death (range)	Mean maximum % wt loss (range)	Median no. of days with indicated wt loss (range)	
						>10%	>20%
NS1-WT	10.8	2	100 (15/15)	NA	8.1 (0.4 to 17.4)	0 (0 to 3)	0 (0)
		10	65 (13/20)	11 (10 to 11)	20.5 (2.5 to >25)	5 (0 to 8)	2 (0 to 7)
		50	15 (3/20)	9 (8 to 10)	24.6 (19.5 to >25)	10 (4 to 10)	8 (0 to 9)
		250	0 (0/5)	9 (8 to 9)	>25 (>25)	10 (10 to 11)	9 (9)
		1,250	0 (0/5)	7 (7 to 8)	>25 (>25)	12 (11 to 12)	10 (9 to 10)
NS1-I106M	4.22	2	100 (15/15)	NA	13.5 (1.3 to 21.8)	2 (0 to 4)	0 (0 to 2)
		10	20 (4/20)	10 (9 to 11)	22.8 (2.2 to >25)	8.5 (0 to 10)	7 (0 to 8)
		50	0 (0/20)	8 (8 to 9)	>25 (>25)	10 (10 to 11)	9 (8 to 9)
		250	0 (4/4)	7.5 (7 to 8)	>25 (>25)	11 (11)	9.5 (9 to 10)
		1,250	0 (5/5)	7 (6 to 7)	>25 (>25)	12 (11 to 12)	10 (10 to 11)

a NA, not applicable.

To assess replication *in vivo*, mice were intranasally infected with 500 PFU of each virus, and lungs were excised on days 2 and 4 postinfection. Following homogenization and centrifugation (10,000 × *g*, 5 min, 4°C), the supernatants were used to determine the viral titer. The NS1-I106M virus replicated to titers >5-fold higher than those of the WT by day 2 (*P* = 0.0056), while titers at day 4 were similar (Fig. 3F). Notably, qRT-PCR analysis of lung homogenates from independently infected mice suggested a trend for the NS1-I106M virus to induce less IFN-β mRNA than the WT virus; however, this difference was not statistically significant (Fig. 3G). These data indicate that, in contrast to *in vitro* results, gain-of-function substitutions in NS1 that enhance CPSF30 binding and inhibition of general gene expression slightly enhance the replication and pathogenicity of H7N9-based viruses *in vivo*.

### Concluding remarks.

Continued zoonotic transmission of H7N9 to humans is a significant cause for concern given the mild to lethal human respiratory disease the virus causes and the fear that it may yet acquire human-to-human transmission capability. Here, we characterize the H7N9 NS1 protein as an efficient IFN antagonist. Nevertheless, H7N9 NS1 is defective in binding CPSF30 and is consequently unable to block host cell gene expression. We identify the single I106M natural polymorphism found in non-H7N9 strains as a potential gain-of-function mechanism by which the H7N9 NS1 could acquire CPSF30 binding and provide evidence that this substitution promotes virus replication and virulence *in vivo*. These results parallel those found with the 1997 H5N1 virus and the laboratory strain PR8, where similar substitutions enhanced CPSF30 binding and virulence (23, 26, 28). Although polymorphisms in H7N9 NS1 that might restore CPSF30 binding have yet to be identified in the sequenced strains that are available, our study highlights the importance of continued surveillance to monitor potential natural gain-of-function mutations in H7N9 NS1 that may impact pathogenicity.

## References

[1] Center for Infectious Disease Research and Policy. 2014 H7N9 avian influenza: resources and literature. Center for Infectious Disease Research and Policy, University of Minnesota, Minneapolis, MN http://www.cidrap.umn.edu/infectious-disease-topics/h7n9-avian-influenza#literature Accessed June 2014

[2] Mok CK Lee HH Lestra M Nicholls JM Chan CW Sia SF Zhu H Poon LL Guan Y Peiris JM 2014 Amino acid substitutions in polymerase basic protein 2 gene contributes to the pathogenicity of the novel A/H7N9 influenza virus in mammalian hosts. J. Virol. 88:3568–3576. 10.1128/JVI.02740-13 24403592 PMC3957932

[3] Zhang H Li X Guo J Li L Chang C Li Y Bian C Xu K Chen H Sun B 2014 The PB2 E627K mutation contributes to the high polymerase activity and enhanced replication of H7N9 influenza virus. J. Gen. Virol. 95(Part 4):779–786. 10.1099/vir.0.061721-0 24394699

[4] Yamayoshi S Yamada S Fukuyama S Murakami S Zhao D Uraki R Watanabe T Tomita Y Macken C Neumann G Kawaoka Y 2014 Virulence-affecting amino acid changes in the PA protein of H7N9 influenza A viruses. J. Virol. 88:3127–3134. 10.1128/JVI.03155-13 24371069 PMC3957961

[5] Gao R Cao B Hu Y Feng Z Wang D Hu W Chen J Jie Z Qiu H Xu K Xu X Lu H Zhu W Gao Z Xiang N Shen Y He Z Gu Y Zhang Z Yang Y Zhao X Zhou L Li X Zou S Zhang Y Li X Yang L Guo J Dong J Li Q Dong L Zhu Y Bai T Wang S Hao P Yang W Zhang Y Han J Yu H Li D Gao GF Wu G Wang Y Yuan Z Shu Y 2013 Human infection with a novel avian-origin influenza A (H7N9) virus. N. Engl. J. Med. 368:1888–1897. 10.1056/NEJMoa1304459 23577628

[6] Kageyama T Fujisaki S Takashita E Xu H Yamada S Uchida Y Neumann G Saito T Kawaoka Y Tashiro M 2013 Genetic analysis of novel avian A(H7N9) influenza viruses isolated from patients in China, February to April 2013. Euro Surveill. 18:20453 http://www.eurosurveillance.org/ViewArticle.aspx?ArticleId=20453 23594575 PMC6296756

[7] Ramos I Krammer F Hai R Aguilera D Bernal-Rubio D Steel J García-Sastre A Fernandez-Sesma A 2013 H7N9 influenza viruses interact preferentially with alpha2,3-linked sialic acids and bind weakly to alpha2,6-linked sialic acids. J. Gen. Virol. 94:2417–2423. 10.1099/vir.0.056184-0 23950563 PMC3809111

[8] Shi Y Zhang W Wang F Qi J Wu Y Song H Gao F Bi Y Zhang Y Fan Z Qin C Sun H Liu J Haywood J Liu W Gong W Wang D Shu Y Wang Y Yan J Gao GF 2013 Structures and receptor binding of hemagglutinins from human-infecting H7N9 influenza viruses. Science 342:243–247. 10.1126/science.1242917 24009358

[9] Xiong X Martin SR Haire LF Wharton SA Daniels RS Bennett MS McCauley JW Collins PJ Walker PA Skehel JJ Gamblin SJ 2013 Receptor binding by an H7N9 influenza virus from humans. Nature 499:496–499. 10.1038/nature12372 23787694

[10] Xu R de Vries RP Zhu X Nycholat CM McBride R Yu W Paulson JC Wilson IA 2013 Preferential recognition of avian-like receptors in human influenza A H7N9 viruses. Science 342:1230–1235. 10.1126/science.1243761 24311689 PMC3954636

[11] Hale BG Randall RE Ortin J Jackson D 2008 The multifunctional NS1 protein of influenza A viruses. J. Gen. Virol. 89:2359–2376. 10.1099/vir.0.2008/004606-0 18796704

[12] Hale BG Steel J Medina RA Manicassamy B Ye J Hickman D Hai R Schmolke M Lowen AC Perez DR García-Sastre A 2010 Inefficient control of host gene expression by the 2009 pandemic H1N1 influenza A virus NS1 protein. J. Virol. 84:6909–6922. 10.1128/JVI.00081-10 20444891 PMC2898253

[13] Hayman A Comely S Lackenby A Murphy S McCauley J Goodbourn S Barclay W 2006 Variation in the ability of human influenza A viruses to induce and inhibit the IFN-beta pathway. Virology 347:52–64. 10.1016/j.virol.2005.11.024 16378631

[14] Kochs G García-Sastre A Martínez-Sobrido L 2007 Multiple anti-interferon actions of the influenza A virus NS1 protein. J. Virol. 81:7011–7021. 10.1128/JVI.02581-06 17442719 PMC1933316

[15] Kuo RL Zhao C Malur M Krug RM 2010 Influenza A virus strains that circulate in humans differ in the ability of their NS1 proteins to block the activation of IRF3 and interferon-beta transcription. Virology 408:146–158. 10.1016/j.virol.2010.09.012 20934196 PMC2975781

[16] Rajsbaum R Albrecht RA Wang MK Maharaj NP Versteeg GA Nistal-Villán E García-Sastre A Gack MU 2012 Species-specific inhibition of RIG-I ubiquitination and IFN induction by the influenza A virus NS1 protein. PLoS Pathog. 8:e1003059. 10.1371/journal.ppat.1003059 23209422 PMC3510253

[17] Barber MR Aldridge JR Jr Webster RG Magor KE 2010 Association of RIG-I with innate immunity of ducks to influenza. Proc. Natl. Acad. Sci. U. S. A. 107:5913–5918. 10.1073/pnas.1001755107 20308570 PMC2851864

[18] Mibayashi M Martínez-Sobrido L Loo YM Cárdenas WB Gale M Jr García-Sastre A 2007 Inhibition of retinoic acid-inducible gene I-mediated induction of beta interferon by the NS1 protein of influenza A virus. J. Virol. 81:514–524. 10.1128/JVI.01265-06 17079289 PMC1797471

[19] Knepper J Schierhorn KL Becher A Budt M Tonnies M Bauer TT Schneider P Neudecker J Ruckert JC Gruber AD Suttorp N Schweiger B Hippenstiel S Hocke AC Wolff T 2013 The novel human influenza A(H7N9) virus is naturally adapted to efficient growth in human lung tissue. mBio 4:e00601–13. 10.1128/mBio.00601-13 24105764 PMC3791893

[20] Das K Ma LC Xiao R Radvansky B Aramini J Zhao L Marklund J Kuo RL Twu KY Arnold E Krug RM Montelione GT 2008 Structural basis for suppression of a host antiviral response by influenza A virus. Proc. Natl. Acad. Sci. U. S. A. 105:13093–13098. 10.1073/pnas.0805213105 18725644 PMC2522260

[21] Nemeroff ME Barabino SM Li Y Keller W Krug RM 1998 Influenza virus NS1 protein interacts with the cellular 30 kDa subunit of CPSF and inhibits 3′end formation of cellular pre-mRNAs. Mol. Cell 1:991–1000. 10.1016/S1097-2765(00)80099-4 9651582

[22] Noah DL Twu KY Krug RM 2003 Cellular antiviral responses against influenza A virus are countered at the posttranscriptional level by the viral NS1A protein via its binding to a cellular protein required for the 3′ end processing of cellular pre-mRNAS. Virology 307:386–395. 10.1016/S0042-6822(02)00127-7 12667806

[23] Twu KY Kuo RL Marklund J Krug RM 2007 The H5N1 influenza virus NS genes selected after 1998 enhance virus replication in mammalian cells. J. Virol. 81:8112–8121. 10.1128/JVI.00006-07 17522219 PMC1951328

[24] Forbes NE Ping J Dankar SK Jia JJ Selman M Keleta L Zhou Y Brown EG 2012 Multifunctional adaptive NS1 mutations are selected upon human influenza virus evolution in the mouse. PLoS One 7:e31839. 10.1371/journal.pone.0031839 22363747 PMC3283688

[25] Ping J Keleta L Forbes NE Dankar S Stecho W Tyler S Zhou Y Babiuk L Weingartl H Halpin RA Boyne A Bera J Hostetler J Fedorova NB Proudfoot K Katzel DA Stockwell TB Ghedin E Spiro DJ Brown EG 2011 Genomic and protein structural maps of adaptive evolution of human influenza A virus to increased virulence in the mouse. PLoS One 6:e21740. 10.1371/journal.pone.0021740 21738783 PMC3128085

[26] Spesock A Malur M Hossain MJ Chen LM Njaa BL Davis CT Lipatov AS York IA Krug RM Donis RO 2011 The virulence of 1997 H5N1 influenza viruses in the mouse model is increased by correcting a defect in their NS1 proteins. J. Virol. 85:7048–7058. 10.1128/JVI.00417-11 21593152 PMC3126612

[27] Dankar SK Miranda E Forbes NE Pelchat M Tavassoli A Selman M Ping J Jia J Brown EG 2013 Influenza A/Hong Kong/156/1997(H5N1) virus NS1 gene mutations F103L and M106I both increase IFN antagonism, virulence and cytoplasmic localization but differ in binding to RIG-I and CPSF30. Virol. J. 10:243. 10.1186/1743-422X-10-243 23886034 PMC3733596

[28] Steidle S Martínez-Sobrido L Mordstein M Lienenklaus S García-Sastre A Staheli P Kochs G 2010 Glycine 184 in nonstructural protein NS1 determines the virulence of influenza A virus strain PR8 without affecting the host interferon response. J. Virol. 84:12761–12770. 10.1128/JVI.00701-10 20926573 PMC3004359

[29] Gack MU Albrecht RA Urano T Inn KS Huang IC Carnero E Farzan M Inoue S Jung JU García-Sastre A 2009 Influenza A virus NS1 targets the ubiquitin ligase TRIM25 to evade recognition by the host viral RNA sensor RIG-I. Cell Host Microbe 5:439–449. 10.1016/j.chom.2009.04.006 19454348 PMC2737813

[30] Hai R Schmolke M Leyva-Grado VH Thangavel RR Margine I Jaffe EL Krammer F Solórzano A García-Sastre A Palese P Bouvier NM 2013 Influenza A(H7N9) virus gains neuraminidase inhibitor resistance without loss of in vivo virulence or transmissibility. Nat. Commun. 4:2854 24326875 10.1038/ncomms3854 PMC3863970

[31] Hai R Martinez-Sobrido L Fraser KA Ayllon J García-Sastre A Palese P 2008 Influenza B virus NS1-truncated mutants: live-attenuated vaccine approach. J. Virol. 82:10580–10590. 10.1128/JVI.01213-08 18768976 PMC2573209

[32] Medina RA García-Sastre A 2011 Influenza A viruses: new research developments. Nat. Rev. Microbiol. 9:590–603. 10.1038/nrmicro2613 21747392 PMC10433403

[33] Kuo RL Krug RM 2009 Influenza a virus polymerase is an integral component of the CPSF30-NS1A protein complex in infected cells. J. Virol. 83:1611–1616. 10.1128/JVI.01491-08 19052083 PMC2643760

